# Virus Genotype Distribution and Virus Burden in Children and Adults Hospitalized for Norovirus Gastroenteritis, 2012–2014, Hong Kong

**DOI:** 10.1038/srep11507

**Published:** 2015-06-17

**Authors:** Martin C.W. Chan, Ting F. Leung, Tracy W.S. Chung, Angela K. Kwok, E. Anthony S. Nelson, Nelson Lee, Paul K.S. Chan

**Affiliations:** 1 Department of Microbiology, Faculty of Medicine, The Chinese University of Hong Kong, Hong Kong Special Administrative Region, People’s Republic of China; 2 Department of Paediatrics, Faculty of Medicine, The Chinese University of Hong Kong, Hong Kong Special Administrative Region, People’s Republic of China; 3 Department of Medicine and Therapeutics, Faculty of Medicine, The Chinese University of Hong Kong, Hong Kong Special Administrative Region, People’s Republic of China

## Abstract

We conducted a 2-year hospital-based study on norovirus gastroenteritis among children and adults between August 2012 and September 2014. A total of 1,146 norovirus cases were identified. Young children (aged ≤ 5 years) accounted for a majority (53.3%) of cases. Hospitalization incidence exhibited a U-shaped pattern with the highest rate in young children (1,475 per 100,000 person-years), followed by the elderly aged > 84 years (581 per 100,000 person-years). A subset (n = 395, 34.5%) of cases were selected for norovirus genotyping and noroviral load measurement. Non-GII.4 infections were more commonly observed in young children than in older adults (aged > 65 years) (20.5% versus 9.2%; p < 0.05). In young children, the median noroviral load of GII.4 and non-GII.4 cases was indistinguishably high (cycle threshold value, median [interquartile range]: 16.6 [15.2–19.3] versus 16.6 [14.9–21.6]; p = 0.45). Two age-specific non-GII.4 genotypes (GII.3 and GII.6) were identified among young children. These findings may have implications in norovirus vaccination strategy.

Noroviruses are a large group of viruses associated with non-bacterial acute gastroenteritis in community and healthcare settings worldwide affecting all age groups^1^. Noroviruses are classified into at least 5 genogroups (GI–GV) and nearly 40 genotypes, in which GI, GII, and GIV infect humans. Despite the genetic diversity, cross-reactive immune responses following norovirus infections have been demonstrated^2^,^3^. Yet, indirect evidence from a recent case report of sequential natural infections of norovirus GII.4 and GII.6 genotypes suggests that at least some genotypes may represent distinct serotype and induce only limited cross-reactive immune response^4^. One particular genotype, known as genogroup II genotype 4 (GII.4), has long been recognized as a leading circulating genotype that causes acute gastroenteritis in developed countries since mid-1990s^5^,^6^. While generally regarded as mild infections in otherwise healthy individuals, norovirus gastroenteritis has been estimated to result in over 14,000 hospitalizations and nearly 1 million health care visits in the United States^7^ and almost 200,000 deaths in developing countries^1^ among young children per year. These figures suggest the need for a norovirus vaccine which is currently under development^8^,^9^. Yet, the broad genetic and probably serotypic diversity of norovirus complicates selection of candidate vaccine strain(s). Studies on the diversity of circulating virus genotypes and the associated virus burden in hospitalized cases of norovirus gastroenteritis that represent the more severe end of the spectrum of disease have been limited^10^,^11^,^12^. The need for these information to assess the disease burden attributed to different norovirus genotypes is warranted. Our group has been conducting hospital-based study on norovirus gastroenteritis since 2012^12^. Here, we reported our latest findings on the genetic diversity and virus burden in hospitalized norovirus gastroenteritis.

## Results

### Description of Norovirus Cases

**Figure 1:**
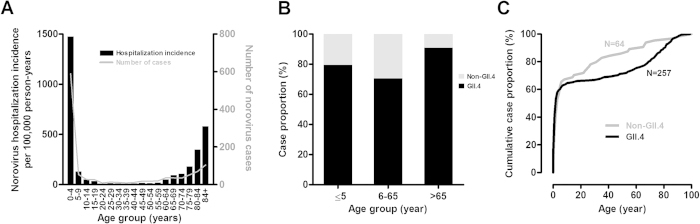
Age distribution of norovirus gastroenteritis. (**A**) Age-stratified hospitalization incidence and number of cases of norovirus gastroenteritis. (**B**) Proportion of norovirus genogroup II genotype 4 (GII.4) and non-GII.4 cases in each age group. (**C**) Age distribution of GII.4 and non-GII.4 cases shown as cumulative case percentage. N, number; yr, year.

A total of 1,146 norovirus cases were identified during the 2-year study period. Nine (0.8%) cases had re-infections and all but 3 were young children aged ≤ 5 years. The male-to-female ratio was 1.11. Age distribution of cases is shown in Fig. 1A. The median age was 4 years (interquartile range [IQR], 1–66 years). Young children aged ≤ 5 years accounted for a majority (53.3%; 611/1,146) of cases. The age-stratified hospitalization incidence exhibited a U-shaped pattern with the highest rate in young children aged < 5 years (1,475 per 100,000 person-years), followed by the elderly aged > 84 years (581 per 100,000 person-years); adults aged 20–59 years were minimally affected (≤20 cases per 100,000 person-years) (Fig. 1A).

### Distribution of Norovirus Genotypes

#### All Selected Cases

A total of 395 (34.5%) cases were selected for noroviral load measurement and genotyping. The age distribution of the selected cases was highly comparable with that of all cases (median [IQR]: 3 [1–60] versus 4 [1–66], years; p = 0.13, Mann-Whitney *U* test). Noroviral load was determined. Norovirus genotype was successfully determined in 321 (81.3%) cases. Failed genotyping was associated with lower noroviral load (Ct value, median [IQR]: 27.8 [24.2–30.8] versus 17.8 [15.8–20.8]; p < 0.0001, Mann-Whitney *U* test). By age, young children aged ≤ 5 years exhibited the highest successful rate of genotyping (86.7%; 195/225), followed by older adults aged > 65 years (77.4%; 65/84) and lowest in older children and adults aged 6–65 years (70.9%; 61/86). Norovirus genotyping successful rate tended to be higher in young children aged ≤5 years than in older adults aged > 65 years (p = 0.055; Fisher’s exact test). Two hundred and fifty seven (80.0%; 257/321) cases were genotyped as GII.4 in which all but four (98.4%) cases belonged to the Sydney 2012 strain. There were 2 cases of GII.4 Den Haag 2006 and 2 cases of GII.4 New Orleans 2009. Among the 64 non-GII.4 cases, 7 (11%) belonged to GI and 58 (91%) to GII with two cases of inter-genogroup and intra-genogroup coinfection. The 3 most common non-GII.4 genotypes were GII.6 (5.9%; 19/321), GII.3 (4.7%; 15/321), and GII.8 (1.6%; 5/321). A list of norovirus non-GII.4 genotype detected in this study is shown in Table 1.

#### Young Children and Older Adults

Among cases of known genotype, GII.4 strains accounted for 79.5% (155/195) of cases in young children aged ≤ 5 years. In contrast, 90.8% (59/65) of cases in older adults aged >65 years were caused by a GII.4 strain (Fig. 1B). Non-GII.4 infections were more commonly observed in young children aged ≤ 5 years than in older adults aged > 65 years (20.5% versus 9.2%; p < 0.05, Fisher’s exact test). In both GII.4 and non-GII.4 infections, a majority of cases were from young children aged ≤5 years (GII.4: 60.3% [155/257]; non-GII.4: 63% [40/64]) (Fig. 1C). Greater norovirus genotype diversity was observed in young children aged ≤ 5 years than in older adults aged >65 years. In young children aged ≤ 5 years, 10 genotypes were detected among the 40 non-GII.4 cases and nearly 70% of cases were caused by GII.6 and GII.3 strains (Table 1). In older adults aged > 65 years, 4 genotypes were detected among 6 non-GII.4 cases (Table 1).

### Comparison of Noroviral Load

#### All Genotyped Cases

**Figure 2:**
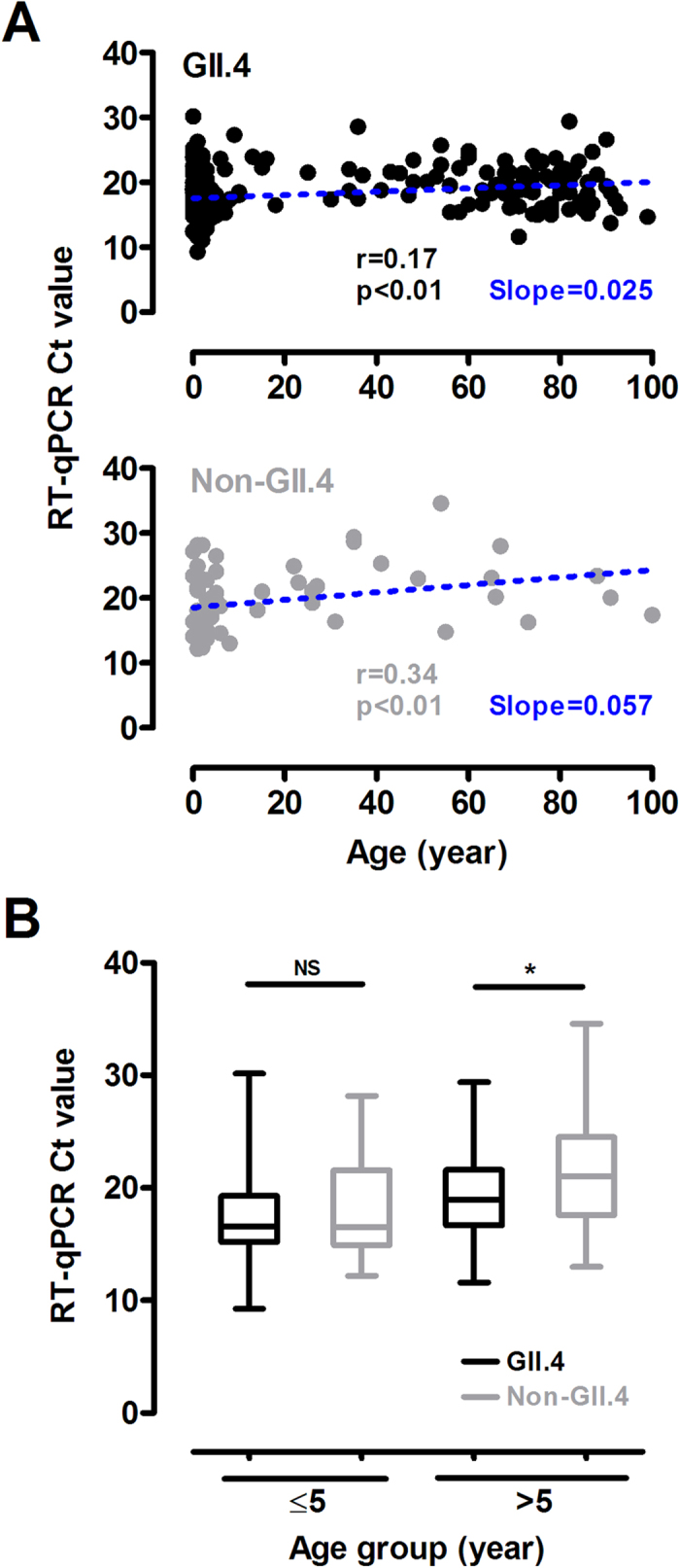
Fecal viral load of norovirus genogroup II genotype 4 (GII.4) and non-GII.4 gastroenteritis on case presentation by (**A**) age and (**B**) age group. Cycle threshold (Ct) value of reverse transcription–polymerase chain reaction (RT-qPCR) assay was determined for each case to represent noroviral load. Higher Ct values indicate lower noroviral loads. Blue dotted lines indicate best-fit lines. In panel **B**, for clarity only p-values (Mann-Whitney *U* test) of comparisons between GII.4 and non-GII.4 cases were shown. Other comparisons were described in the main text where appropriate. Red horizontal lines denote medians. NS, not significant; r, Spearman’s rank correlation coefficient; *, p < 0.05.

The distribution of noroviral load on presentation with age in GII.4 and non-GII.4 cases was shown in Fig. 2A. In general, noroviral load decreased with increasing age (GII.4: r = 0.17, p < 0.01; non-GII.4: r = 0.34, p < 0.01; Spearman correlation). The slope of the best-fit line of non-GII.4 cases (0.057) was greater than that of GII.4 cases (0.025), indicating the decline in noroviral load with age was more pronounced in non-GII.4 cases. The median noroviral load of GI cases was over 1,000-fold lower than that of other non-GII.4 cases (Ct value, median [IQR]: 28.0 [25.0–30.7] versus 17.4 [15.0–21.8]; p < 0.001, Mann-Whitney *U* test).

#### Young Children and Older Adults

Noroviral load by age group is shown in Fig. 2B. Highest median noroviral load was observed in young children aged ≤ 5 years. In GII.4 infections, the median noroviral load in young children aged ≤ 5 years was ~5-fold higher than that in case aged > 5 years (Ct value, median [IQR]: 16.6 [15.2–19.3] versus 19.0 [16.7–21.6]; p < 0.0001, Mann-Whitney *U* test). In non-GII.4 infections, the median noroviral load in young children aged ≤ 5 years was ~23-fold higher than that in cases aged > 5 years (Ct value, median [IQR]: 16.6 [14.9–21.6] versus 21.1 [17.6–24.5]; p < 0.05, Mann-Whitney *U* test). In young children aged ≤5 years, the median noroviral load of GII.4 and non-GII.4 cases was indistinguishably high (Ct value: 16.6 versus 16.6; p = 0.45, Mann-Whitney *U* test). In cases aged > 5 years, the median noroviral load of non-GII.4 cases was ~4-fold lower than that of GII.4 cases (Ct value: 21.1 versus 19.0; p < 0.05, Mann-Whitney *U* test). When analyzing older children and adults aged 6–65 years and older adults aged >65 years separately, the median noroviral load of non-GII.4 cases was consistently lower than that of GII.4 cases, although the difference did not reach statistical significance.

### Characteristics of Norovirus GII.6, GII.3 and GII.8 Infections

Norovirus GII.6, GII.3, and GII.8 strains were the 3 most common non-GII.4 genotypes detected in our study. Their age distribution and fecal noroviral load are shown in Table 1. The median age of GII.6 and GII.3 cases were 2 years (IQR: 1–4 years) and 1 year (IQR: 1–4 year), respectively. Young children aged ≤5 years accounted for 79% (15/19) and 80% (12/15) of GII.6 and GII.3 cases, respectively. In contrast, the median age of GII.8 cases was 88 years (IQR: 27–96 years). No GII.8 cases were observed in young children aged ≤5 years. The median age of the 5 GII.8 cases was higher than that of GII.3 and GII.6 cases (both p < 0.01; Kruskal-Wallis test). The median noroviral load of GII.8 (Ct value: 20.1) cases was lower than that of GII.6 (Ct value: 16.8) and GII.3 (Ct value: 16.6) but the difference did not reach statistical significance.

### Norovirus Re-infections

There were 9 cases of re-infection and 2 cases were genotyped. The first genotyped case was a one-year-old girl. Her first episode of gastroenteritis was attributed to a GII.6 strain and the second episode was caused by a GII.4 Sydney 2012 strain separated by a 4-month period. Both stool specimens showed high noroviral loads (Ct values: 18.1 [GII.6] and 20.1 [GII.4]). The second genotyped case was a male infant aged <1 year. He was first infected with a GII.4 Sydney 2012 strain with high virus shedding (Ct value: 16.0) and was admitted again for norovirus gastroenteritis after 1 month. The noroviral load of the second episode was too low for virus genotyping (Ct value: 33.3).

## Discussion

Studies on hospitalization of norovirus gastroenteritis have focused on either young children or older adults^7^,^10^,^13^,^14^,^15^. Our study, to the best of our knowledge, is comprehensive in investigating genetic diversity and virus burden of norovirus gastroenteritis in both children and adults in a hospital setting. There are three key observations: (1) young children aged ≤5 years accounted for a majority (>60%) of norovirus GII.4 and non-GII.4 infections requiring hospitalization, extending the findings of our previous study^12^; (2) non-GII.4 genotypes accounted for 20.5% and 9.2% of hospitalized cases of young children aged ≤5 years and older adults aged >65 years, respectively; and (3) both GII.4 and non-GII.4 infections exhibited indistinguishably high noroviral load (both with median Ct values of 16.6) in young children aged ≤5 years. It has long been recognized that there is a high degree of norovirus genotype diversity in young children^16^,^17^. With the recent advancement in the development of monovalent and bivalent norovirus vaccines^8^,^18^,^19^, one important unanswered question is whether to include a second genotype together with the contemporarily predominant GII.4 genotype as a vaccine component. Our findings that non-GII.4 infections attained virtually identical high noroviral load as in GII.4 infections in young children clearly indicates that non-GII.4 genotypes are replication competent in young children. In our study, norovirus GII.6 and GII.3 genotypes are common in young children which is in agreement with other epidemiological studies^11^,^14^. Notably, studies on historical stool specimens dated back to the 1970 s and 1980 s suggested that GII.3, rather than GII.4, was the main circulating norovirus genotype at that time, at least in hospitalized pediatric diarrheal cases^20^. This suggests that non-GII.4 genotypes such as GII.3 may be a candidate vaccine genotype. While a monovalent GII.4 vaccine could cover a large majority of hospitalized norovirus infections, a multivalent vaccine supplemented with circulating non-GII.4 genotype(s) may offer additional protection, especially for young children and should be considered. Notably, immune escape norovirus GII.4 strains emerged every 2 to 4 years. Our study was performed soon after the emergence of the then novel GII.4 Sydney 2012 strain which remained as the major circulating strain throughout the study period. This allows us to explore how emergent GII.4 strains influence the prevalence and seasonality of non-GII.4 genotypes. Continuous epidemiological surveillance of non-GII.4 infections is necessary as the herd immunity of non-GII.4 genotypes may influence norovirus circulating patterns^21^.

The duration and cross-protection of norovirus immunity have been topics of hot debate. Experimental volunteer challenge study by a GI strain known as Norwalk virus suggests that homotypic immunity to norovirus infection may last for 8 weeks to 2 years^22^. A much longer duration of immunity from 4 to 8 years has been estimated from mathematical modeling that used population-based incidence data^23^. In our 2-year study, norovirus re-infections that required hospitalization were rarely observed. This is consistent with the notion that norovirus immunity may endure 2 years or longer. It is possible that we might have missed some cases of norovirus re-infections if the second episode of gastroenteritis was not severe enough to trigger medical attention and hospitalization as a result of partial immunity. In a recent longitudinal community-based follow-up study of a birth cohort in Peru, multiple infections by different norovirus genotypes were commonly observed in nearly half of cases^24^. Re-infections may be more likely to be detected in setting of milder norovirus gastroenteritis. We genotyped two paediatric cases of norovirus re-infection in which one case was confirmed to be infected with two different genotypes (GII.6 and GII.4) within a short period of 4 months. This suggests that protective heterotypic immunity may not occur following natural infection^4^. Interestingly, noroviral loads of both GII.4 and non-GII.4 cases decreased with age. One interpretation is that previous norovirus exposure may confer partial immunity which is limited in strength. Alternatively, longer delay in seeking medical care in older patients may confound noroviral load data.

Our study has limitations. Firstly, we did not determine patients’ Vesikari score to evaluate whether norovirus GII.4 and non-GII.4 infections in young children lead to similar clinical severity of gastroenteritis despite their similarly high viral loads that has been shown to associate with longer duration of diarrhea^25^,^26^. Huhti and colleagues have shown that the GII.4 genotype was associated with more protracted diarrhea and vomiting than non-GII.4 genotypes^27^. It should be, however, noted that in the study of Huhti the predominant non-GII.4 genotypes were GII.b (now known as GII.P21/GII.3), GII.2, and GII.7 instead of GII.6 and GII.3 as in our study. There is a possibility that different norovirus genotypes display distinct clinical characteristics. Likewise, it is also elusive whether differential viral loads among different age groups are associated with clinical severity. Secondly, non-GII.4 cases aged >5 years had the lowest noroviral load. This may lead to underestimation of the prevalence of non-GII.4 cases in older cases as genotyping is more likely to fail due to insufficient noroviral load. Thirdly, it is unclear whether our findings can be generalized to other settings such as community settings and foodborne outbreaks^5^,^28^,^29^. Nevertheless, hospitalized cases are likely to represent more severe infections among all-cause norovirus gastroenteritis and they are one of main indexes in evaluating efficacy of a vaccine and success of a vaccination program.

In summary, among hospitalized cases of norovirus gastroenteritis, non-GII.4 infections are common in young children, usually with high virus burden, and are not uncommon in older adults. Both norovirus GII.4 and non-GII.4 infections exhibit indistinguishably high virus burden in young children. There may be a need for a multivalent norovirus vaccine targeted to young children and probably older adults who will most likely experience severe infections in the future. Continuous epidemiological surveillance of non-GII.4 infections is necessary to guide the selection of norovirus vaccine strain.

## Materials and Methods

### Catchment Population and Case Selection

This is a 2-year hospital-based study on norovirus gastroenteritis conducted between August 2012 and September 2014. Our study site, the Prince of Wales Hospital, is the norovirus diagnosis laboratory for Hospital Authority’s New Territories East Cluster that comprises of 3 major acute general hospitals, serving a population of about 1.2 million and covering nearly 20% of entire population in Hong Kong. Stool specimens were collected from patients admitted with acute gastroenteritis (diarrhea [loose/watery stool] or vomiting with a frequency of 3 or more in 24 hours) and tested for norovirus upon clinical suspicion (without other alternative explanation such as underlying bowel disorder) of norovirus infections. Diagnostic testing was performed twice weekly in batches. Patients tested positive for norovirus were identified. For this study, a subset of norovirus cases were selected for noroviral load measurement and genotyping as follows: during August 2012–February 2014, all consecutive cases tested positive for norovirus in the first test batch of each week were selected; during March 2014–September 2014, all consecutive cases tested positive in both weekly batches were selected as part of enhanced surveillance for emergent norovirus GII.4 strain. Cases with norovirus detected in separate occasions with more than 30 days apart were regarded as having re-infections. Patients’ demographic information of age and sex were retrieved from the computerized clinical management system. For calculating age-stratified norovirus hospitalization incidence, the catchment population was used as the at-risk population. Catchment population statistics was compiled from the most recent Hong Kong 2011 Population Census (http://www.census2011.gov.hk/en/district-profiles.html). Ethics approval for this study and waiver of consent of using de-identified leftover diagnostic specimens were obtained from the joint Chinese University of Hong Kong–New Territories East Cluster clinical research ethics committee (reference number CRE-2013.330). All experiments were performed in accordance with relevant guidelines and regulations.

### Norovirus Detection and Noroviral Load Quantification

Viral RNA was extracted and purified from ~10% (0.1 gram) stool suspension using MagMAX Viral RNA Isolation Kit (Life Technologies). Norovirus detection and noroviral load quantification was performed by using a quantitative, genogroup-specific, probe-based real-time reverse transcription–polymerase chain reaction (RT-qPCR) assay that targeted the junction between RNA-dependent RNA polymerase (RdRp) and viral protein 1 (VP1) genes of norovirus as previously described^30^. Briefly, 10 μL of purified RNA was used as template in a 25-μL total reaction volume using SuperScript III Platinum One-Step qRT-PCR kit (Life Technologies). No template (negative) control was included in every run. To control for batch-to-batch variation, a positive control of known Ct value was included in each run. Assay cycle threshold (Ct) value was determined for each case to represent noroviral load. Higher Ct values indicated lower noroviral loads.

### Norovirus Genotyping

Viral RNA was converted into complementary DNA (cDNA) using SuperScript III reverse transcriptase in the presence of random hexamers (Life Technologies). Norovirus genogroup-specific PCR amplification of a short fragment (~500 bp) comprising partial RdRp and VP1 genes of the virus was generated using primers GIFF/G1SKR (for norovirus GI) and G2FB/G2SKR (for norovirus GII)^30^. Viral RNA that failed to generate a PCR product was subjected to a separate round of reverse transcription using SuperScript III reverse transcriptase in the presence of a tagged oligo-dT primer, followed by a genogroup-specific long PCR of the 3’ end of the virus genome that covered VP1 and VP2 genes and 3’-untranslated region using primers COG1F/Tag (for norovirus GI) and COG2F/Tag (for norovirus GII). PCR products were purified by treatment with ExoSAP-IT (Affymetrix) and then directly sequenced by the forward PCR primer using Sanger sequencing. Chromatograms were manually checked using ChromasPro version 1.7.6 (Technelysium). Virus genotype assignment based on the partial VP1 gene was performed using RIVM’s online norovirus typing tool (http://www.rivm.nl/mpf/norovirus/typingtool)^31^. Non-GII.4 genotypes in this study were defined to include all genotypes of GI and GII except GII.4.

### Statistical Analysis

Categorical variables were compared using Fisher’s exact test. Continuous variables were compared using Mann-Whitney *U* test (between 2 groups) or Kruskal-Wallis test with Dunn’s post hoc method to correct for multiple comparison (among 3 groups or more). Correlation between two continuous variables was measured by Spearman correlation. All statistical tests were performed using Prism version 5.04 (GraphPad). A two-tailed p-value < 0.05 was considered to be statistically significant.

## Additional Information

**How to cite this article**: Chan, M. C.W. *et al.* Virus Genotype Distribution and Virus Burden in Children and Adults Hospitalized for Norovirus Gastroenteritis, 2012-2014, Hong Kong. *Sci. Rep.* **5**, 11507; doi: 10.1038/srep11507 (2015).

## Figures and Tables

**Table 1:** Age-stratified norovirus genotype detected in this study and associated viral load.

**Genogroup.Genotype**	**All cases (N** **=** **321)**	**Young children (≤5 yr)(N** **=** **195)**	**Older children and adults(6–65 yr)(N** **=** **61)**	**Older adults (>65 yr)(N** **=** **65)**	**Age [median (IQR)], yr**	**Viral load [median (IQR)], Ct**
Genogroup I						
All GI	6	2	4	−	35 (4–44)	28.0 (25.0–30.7)
GI.2	3	−	3	−	NC	NC
GI.4	1	1	−	−	NC	NC
GI.5	1	-	1	−	NC	NC
GI.8	1	1	−	−	NC	NC
Genogroup II						
GII.4	257	155	43	59	2 (1–60)	17.7 (15.8–20.2)
All non-GII.4 GII	56	37	13	6	3 (1–20)	17.4 (15.0–21.8)
GII.2	3	1	2	−	NC	NC
GII.3	15	12	3	−	1 (1–4)	16.6 (14.9–21.2)
GII.5	2	1	1	−	NC	NC
GII.6	18	15	2	1	2 (1–4)	16.8 (14.8–21.0)
GII.7	3	2	1	−	NC	NC
GII.8	5	−	2	3	88 (27–96)	20.1 (16.9–24.2)
GII.13	2	1	−	1	NC	NC
GII.17	3	1	1	1	NC	NC
GII.21	3	3	−	−	NC	NC
Indeterminant*	2	1	1	−	NC	NC
Co-infection						
GI.2/GII.6	1	−	1	−	NC	NC
GII.13/GII.17	1	1	−	−	NC	NC

Note. Age and viral load of norovirus genotypes with number of cases ≥5 are shown. -, none detected; Ct, cycle threshold; GII.4, genogroup II genotype 4; IQR, interquartile range; N, number; NC, not calculated; yr, year.

^*^Undefined GII genotype from the norovirus typing tool.
